# Amino-acid transporters in T-cell activation and differentiation

**DOI:** 10.1038/cddis.2016.222

**Published:** 2017-03-02

**Authors:** W Ren, G Liu, J Yin, B Tan, G Wu, F W Bazer, Y Peng, Y Yin

**Affiliations:** 1 Key Laboratory of Agro-ecological Processes in Subtropical Region, Institute of Subtropical Agriculture, Chinese Academy of Sciences; Observation and Experiment Station of Animal Nutrition and Feed Science in South-Central China, Ministry of Agriculture; Hunan Provincial Engineering Research Center for Healthy Livestock and Poultry Production, Changsha 410125, China; 2 University of the Chinese Academy of Sciences, Beijing 10008, China; 3 Department of Animal Science, Texas A&M University, 2471 TAMU, College Station, TX 77843-2471, USA; 4 Chongqing Key Laboratory of Forage and Herbivore, College of Animal Science and Technology, Southwest University, Chongqing 400716, China

## Abstract

T-cell-mediated immune responses aim to protect mammals against cancers and infections, and are also involved in the pathogenesis of various inflammatory or autoimmune diseases. Cellular uptake and the utilization of nutrients is closely related to the T-cell fate decision and function. Research in this area has yielded surprising findings in the importance of amino-acid transporters for T-cell development, homeostasis, activation, differentiation and memory. In this review, we present current information on amino-acid transporters, such as LAT1 (l-leucine transporter), ASCT2 (l-glutamine transporter) and GAT-1 (*γ*-aminobutyric acid transporter-1), which are critically important for mediating peripheral naive T-cell homeostasis, activation and differentiation, especially for Th1 and Th17 cells, and even memory T cells. Mechanically, the influence of amino-acid transporters on T-cell fate decision may largely depend on the mechanistic target of rapamycin complex 1 (mTORC1) signaling. These discoveries remarkably demonstrate the role of amino-acid transporters in T-cell fate determination, and strongly indicate that manipulation of the amino-acid transporter-mTORC1 axis could ameliorate many inflammatory or autoimmune diseases associated with T-cell-based immune responses.

## Facts

1. The cellular metabolic pathways are associated with the shaping T-cell development, homeostasis, activation, differentiation and even memory.
2. AA transporters are critical for T-cell fate decision.
3. AA transporters, such as LAT1, ASCT2, and GAT-1, have important roles in peripheral naive T-cell homeostasis, T-cell activation and differentiation, especially for Th1 and Th17 cells, and T-cell memory.
4. The influence of AA transporters on T-cell fate decision may largely depend on mTORC1 signaling.

## Open Questions

1. Besides LAT1, ASCT2, and GAT-1, whether other AA transporters are also expressed in T cells and function in T-cell fate decision, and how do they affect the T-cell fate decision?
2. In addition to mTORC1 signaling, do AA transporters affect T-cell fate decision through other molecular mechanisms?
3. Do AA transporters also affect unconventional T cells, including CD1-restricted T cells, MR1-restricted mucosal-associated invariant T cells, MHC class Ib-reactive T cells and *γδ* T cells?

**Figure 1:**
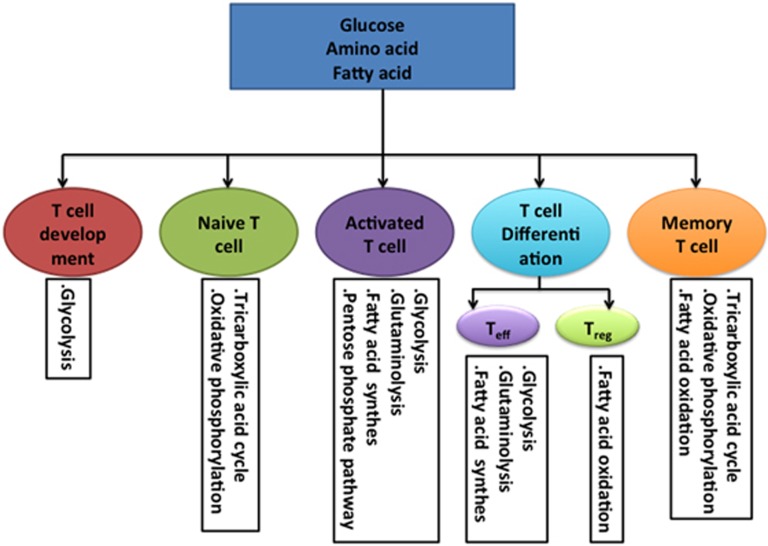
Dominant metabolic pathways in different stages of T cells. Although T cells at all stages can use glucose, AAs and fatty acids, the main metabolic pathways differ depending on the stage of the T cells. Glycolysis is important for T-cell development in the thymus, while most energy for naive T cells is produced in the mitochondria through the fermentation of acetyl-coenzyme A (CoA) in the TCA cycle and OXPHOS. Upon activation, T cells rapidly and massively upregulate the glycolytic, glutaminolytic and pentose phosphate pathways for the production of energy and synthesis of biomass. Also, activated T cells switch from lipid metabolism via *β*-oxidation to fatty acid synthesis. For T-cell differentiation, effector T cells use energy from glucose through glycolysis, and AA through glutaminolysis, whereas regulatory T cells use energy from FAO. Memory T cells mainly use *β*-oxidation of fatty acids to meet their energy needs

T-cell-mediated immune responses are necessary to efficiently protect mammals against infections. To protect the host from infection, naive T cells need to go through the following phases: (a) a beginning phase with massive clonal expansion and differentiation of T cells; (b) a second phase, including the migration of T cells to relevant tissues, synthesis of cytokines and effector molecules, as well as the clearance of most effector cells; and (c) a final phase with the generation of memory T cells. This process imposes considerable demands for energy and biosynthetic precursors.^1^ The uptake and utilization of nutrients highly affects T-cell development, homeostasis, activation, differentiation and memory.^1, 2, 3, 4, 5^ T cells in each stage or even distinct T-cell subsets within a similar stage display unique metabolic programs (Figure 1). For example, naive T cells are quiescent to avoid nonspecific or excess immune reactions, thus their intracellular metabolism is largely dependent on the tricarboxylic acid (TCA) cycle and oxidative phosphorylation (OXPHOS) for the generation of adenosine triphosphate (ATP).^4^ Upon activation, T cells rapidly proliferate and differentiate, and produce various cytokines, all of which require more energy substrates.^6^ Activated T cells fulfill these demands through swift metabolic changes that increase glycolysis, glutaminolysis and fatty acid synthesis.^4^ Also, human T-regulatory cells mainly use fatty acid oxidation (FAO) when proliferating *in vitro*, whereas the proliferation of human T conventional cells mainly depends on the metabolism of glucose.^7^ Thus, there is increasing interest in regulating T-cell fate decision by modulating the abundance of nutrients in cells, expression of nutrient transporters and activation of metabolic pathways, especially for those of glucose and fatty acid.^1, 2, 8^ Amino acids (AA) or AA transporters are also crucial in T-cell-mediated immunity.^9, 10, 11^ For example, activated T cells use glutamine to fuel metabolism, as a nitrogen source and as an anapleurotic substrate.^12^ This review focuses on our current understanding of AA transporters and their significance in the development, differentiation, homeostasis, activation and memory processes of T cells.

## Expression of AA transporters in T cells

**Figure 2:**
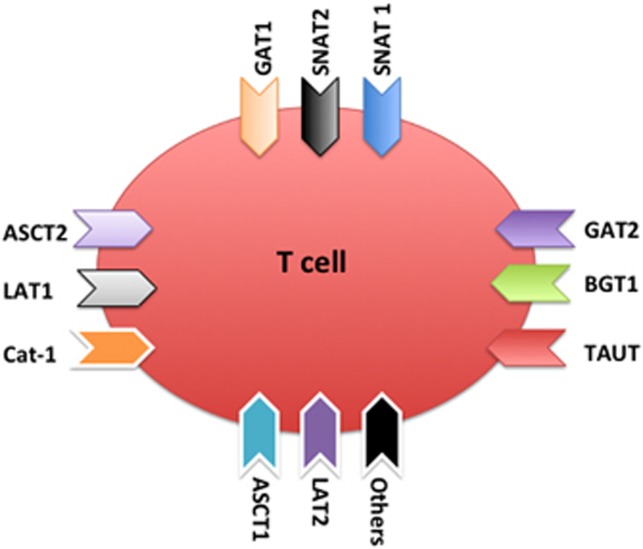
Expression of AA transporters in T cells. Current evidence shows that T cells express SNAT1 (Slc38a1), SNAT2 (Slc38a2), GAT-1 (Slc6a1), GAT-2 (Slc6a13), BGT1 (Slc6a12), TAUT (Slc6a6), ASCT1 (Slc1a4), ASCT2 (Slc1a5), LAT1 (Slc7a5), LAT2 (Slc7a8) and Cat-1 (Slc7a1). Other means that the expression of more AA transporters in T cells needs further investigation

Based on their substrate specificity, transport mechanism and regulatory properties,^13, 14^ AA transporters can be classified as: (1) sodium-dependent neutral AA transporters, including system A [SNAT-1 (Slc38a1), SNAT-2 (Slc38a2), SNAT4 (Slc38a4)], ASC [ASCT1 (Slc1a4), ASCT2 (Slc1a5)], BETA [GAT-1 (Slc6a1), GAT-2 (Slc6a13), GAT3 (Slc6a11), BGT1 (Slc6a12), TAUT (Slc6a6)], Gly [GLYT1 (Slc6a9), GLYT2 (Slc6a5)], N [SNAT3 (Slc38a3), SNAT5 (Slc38a5)] and PRQT [PROT (Slc6a7)] (2) sodium-independent neutral AA transporters, including system asc* [Asc (Slc7a10)], imino [PAT1/LYAAT1 (Slc36a1), PAT2/LYAAT2 (Slc36a2)], L* [LAT1 (Slc7a5), LAT2 (Slc7a8)] and T [TAT1 (Slc16a10)] (3) sodium-dependent anionic AA transporters-system X^−^_AG_ [EAAT1 (Slc1a3), EAAT2 (Slc1a2), EAAT3 (Slc1a1), EAAT4 (Slc1a6), EAAT5 (Slc1a7)] (4) sodium-independent anionic AA transporters system x−^C*^ [xCT (Slc7a11)] (5) sodium-dependent cationic AA transporters, including system B^0,+^ [ATB(0,+) (Slc6a14)] and y^+^L* [y+LAT1 (Slc7a7), y+LAT2 (Slc7a6)] and (6) sodium-independent cationic AA transporters, including system b^0,+**^ [b(0,+)AT (Slc7a9)] and y^+^ [Cat-1 (Slc7a1), Cat-2 (Slc7a2), Cat-3 (Slc7a3), Cat-4 (Slc7a4)]. T cells express various AA transporters (Figure 2). For example, fetal T cells express SNAT-1 and SNAT-2 mRNAs.^15^ T cells express various numbers of the system BETA family, including GAT-1,^9, 16, 17^ GAT-2,^17^ BGT1^18^ and TAUT.^19^ Other AA transporters have also been reported in T cells, such as ASCT2,^10^ LAT1^20^ and Cat-1.^21^

Collectively, although a full expression profile of AA transporters in T cells is missing, current evidence shows that T cells express various types of AA transporters, and AA transporters may affect the T-cell fate decision, including development, homeostasis, activation/differentiation and memory.

## AA transporters and intrathymic development of T cells

**Figure 3:**
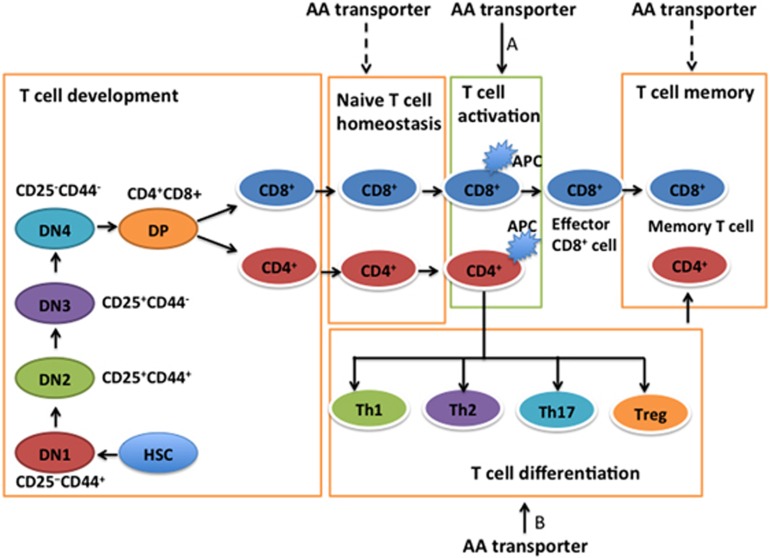
Roles of AA transporters in T-cell fate. The influences of AA transporters on T-cell development, homeostasis, activation/differentiation and memory processes are illustrated here. AA transporters may be needed for the homeostasis of resting T cells and T-cell memory (dashed arrows), whereas AA transporters have critical importance in the activation and differentiation of T cells (black arrows). Conventional T cells experience sequential developmental stages before the emergence of naive resting T cells, including CD4^−^CD8^−^ DN thymocytes, CD4^+^CD8^+^ DP thymocytes and CD4^+^ or CD8^+^ SP thymocytes. CD4^−^CD8^−^ DN thymocytes can be further classified into four sub-populations (DN1–4) based on the expression of CD25 and CD44: DN1 (CD25^−^CD44^+^), DN2 (CD25^+^CD44^+^), DN3 (CD25^+^CD44^−^) and DN4 (CD25^−^CD44^−^). A: LAT1 and ASCT2 promote T-cell activation, whereas GATs inhibit T-cell activation. B: LAT1 and ASCT2 are positively associated with the differentiation of T-helper type 1 and 17 (Th1 and Th17) cells, whereas GAT-1 is negatively associated with differentiation of Th1 and Th17 cells

Major events for the development of T cells in the thymus are well established.^5, 22, 23, 24^ Conventional T cells (T-cell receptor (TCR) *αβ*) experience sequential developmental stages before the emergence of naive resting T cells, including CD4^−^CD8^−^ double-negative (DN) thymocytes, CD4^+^CD8^+^ double-positive (DP) thymocytes and CD4^+^ or CD8^+^ single-positive (SP) thymocytes (Figure 3). CD4^−^CD8^−^ DN thymocytes are further classified into four sub-populations (DN1–4) based on their expression of CD25 and CD44: DN1 (CD25^−^CD44^+^), DN2 (CD25^+^CD44^+^), DN3 (CD25^+^CD44^−^) and DN4 (CD25^−^CD44^−^) (Figure 3).

In the thymus, the development of T cells is highly dependent on the cellular metabolism. Alterations in metabolism-related signals significantly effect T-cell development, such as phosphatidylinositol 3-kinase-Akt, AMP-activated protein kinase and Notch signaling.^3^ However, the AA-tuned mechanistic target of rapamycin complex 1 (mTORC1) has limited influence on T-cell development in the thymus. Mice with *Tsc1* (an inhibitor of mTORC1) deletion in T cells have similar numbers of total thymocytes, DN, DP, CD4^+^ SP and CD8^+^ SP subsets, as well as a comparable expression of thymocyte maturation markers, including CD62L, CD69 and CD24, compared to wild-type (WT) mice.^25^ Similarly, another independent study has found that *Tsc1* deletion in the T cells of mouse has little effect on the total number of thymic cells and the percentages of thymocyte subsets, including DN, DP, CD4^+^ SP and CD8^+^ SP cells in the thymus.^26^ Mice with *Raptor* (an obligate adaptor for mTORC1) deficiency in T cells have similar percentages and numbers of total thymocytes, DN, DP, CD4^+^ SP and CD8^+^ SP subsets, when compared with WT mice.^27^ Interestingly, although mice with T-cell-specific *Raptor* deficiency show little effect on T-cell development, rapamycin (an inhibitor of mTORC1) treatment or *Raptor* deficiency in all tissues of mice induces apparent atrophy of the thymus and inhibits T-cell development.^28^ Rapamycin significantly decreases the percentage of DP cells, but increases the percentage of DN3 cells, suggesting that rapamycin largely blocks DN3 to DP differentiation.^28^ Mice with *Raptor* deficiency in all tissues show a reduction in the absolute numbers of DN2 and DN3 cells, but an increase in the proportion of DN1 cells, indicating that *Raptor* has an important role in the development of early T-cell progenitors, particularly at the DN2 stage.^28^ Thus, mTORC1 signaling may regulate early T-cell development, but may not be critically involved in the late development of T cells.

CD98 (Slc3a2) forms complexes with either Slc7a5, Slc7a8, Slc7a7 or Slc7a6 to form system L* and system y+L AA transporters, which transport leucine and large neutral AA.^29^ Early observations that DN cells express CD98^30^ indicated that some AA transporters might have an important influence on the development of T cells in the thymus. Selective deletion of CD98 in mouse T cells markedly reduces the clonal expansion of T cells,^31^ and mice with CD98 deficiency in T cells can accept a full major histocompatibility complex-mismatched cardiac allograft.^32^ Further research has indicated that CD98 regulates T cells by amplifying integrin signaling, but not because it forms complexes with some AA transporter light chains.^31^ *Slc7a5*^fl/fl^CD4-Cre mice, which have deletion of the *Slc7a5* in DP thymocytes and all subsequent T-cell populations, exhibit normal numbers and frequencies of conventional *αβ* T cells in the thymus.^11^ The *Slc7a5*^fl/fl^Vav-Cre mice with deletion of *Slc7a5* in hematopoietic progenitors in bone marrow have normal thymocyte numbers and distribution of CD4^−^CD8^−^ DN, CD4^+^CD8^+^ DP, CD4^+^ SP and CD8^+^ SP subsets.^11^ Deletion of the *Slc1a5* in mice shows normal thymocyte development compared with WT mice, as revealed by the comparable frequencies of thymocyte sub-populations, including CD4^−^CD8^−^ DN, CD4^+^CD8^+^ DP, CD4^+^ SP, CD8^+^ SP subsets, and similar numbers of total thymocytes in the *Slc1a5*^+/+^ and *Slc1a5*^–/–^ mice.^10^ The *γ*-aminobutyric acid (GABA) transporter-1 (GAT-1) also has a limited role in T-cell development in the thymus based on evidence that *Slc6a1*^−/−^ mice^9^ exhibit similar percentages and numbers of total thymocytes, of CD4^−^CD8^−^ DN and CD4^+^CD8^+^ DP subsets and CD4^+^ SP and CD8^+^ SP subsets, with their controls.

Although understanding of the roles of other AA transporters in T-cell development in the thymus requires further investigation, these present compelling studies indicate a limited importance of AA transporters in T-cell development in the thymus.

## AA transporters and naive T-cell homeostasis

Resting naive T cells are characterized by their small cell size and continuous migration through the secondary lymphoid tissues for immune surveillance. To promote homeostatic growth and survival, naive T cells require ATP generated from the TCA cycle and OXPHOS (Figure 1).^3, 33^ Thus, these cells can use a variety of nutrients to meet metabolic demands, including glucose through glycolysis, AA through glutamine oxidation and lipids through fatty acid *β*-oxidation.^4^ Besides the engagement of TCRs by the self-peptide-MHC complex, the interaction between interleukin-7 (IL-7) and IL-7R is of critical importance to naive T-cell homeostasis, proliferation and prolonged survival.^34, 35^ Although AAs are not required for IL-7-induced survival of naive CD8^+^ T cells, AAs are essential for the maintenance of naive CD8^+^ T-cell size and IL-7-induced growth.^36^ CD8^+^ T cells express mRNAs of several AA transporters, including *Slc1a4*, *Slc1a5*, *Slc7a5* and *Slc7a6*, and IL-7 stimulation increases the transcription of *Slc1a4*, *Slc1a5* and *Slc7a5* in CD8^+^ T cells.^36^ Although the CD4^+ ^: CD8^+^ ratios in the spleen, peripheral blood and lymph nodes, and T-cell percentages in peripheral blood and lymph nodes are similar between WT mice and mice with an mTORC1 deficiency in T cells,^37^ the mTORC1 pathway is a critical factor for the maintenance of quiescence and homeostasis of peripheral T cells because *Tsc*^−/^^−^ T cells,^25, 38^ *Pten*^−^^/^^−^ (phosphatase and tensin homolog, an inhibitor of mTORC1 signaling) T cells^39^ and *Lkb1*^−/−^ (liver kinase B1, an inhibitor of mTORC1 signaling) T cells^40, 41^ lose quiescence and spontaneous entry into the cell cycle and are sensitive to undergoing apoptosis, compared with WT controls. These evidences indicate that AA transporters may have critical roles in the homeostasis of peripheral naive T cells.

Mice with a single *Slc7a5* allele deletion have normal peripheral lymphocyte sub-populations.^11^ *Slc7a5*^fl/fl^CD4-Cre mice show little change in the numbers and frequencies of naive CD4^+^ and CD8^+^ T-cell subsets in the spleen and lymph nodes, and little alteration in the numbers and frequencies of peripheral T-lymphocyte sub-populations.^11^ At young ages (6–7 weeks), *Slc1a5*^–/–^ mice have similar numbers of T cells, naive CD4^+^ T cells and naive CD8^+^ T cells in the spleen, compared to the *Slc1a5*^+/+^ mice.^10^ However, older *Slc1a5*^–/–^ mice (5–6 months) have a reduced percentage and number of CD4^+^ T cells, while there is an increased frequency of the CD44^lo^CD62L^hi^-naive CD4^+^ T cells compared with *Slc1a5*^+/+^ mice.^10^ These results suggest that AA transporters have few roles in maintaining peripheral naive T-cell homeostasis in young mice, but may affect naive T-cell homeostasis in older mice. A deficiency of GAT-1 in a mouse does not affect naive T-cell homeostasis because *Slc6a1*^−/−^ mice have similar numbers of CD4^+^ cells, CD8^+^ cells and similar ratios of T cells/B cells and CD4^+^ cells/CD8^+^ cells in the spleen as for WT mice.^9^

Collectively, these studies suggest that AA transporters have little effect on the homeostasis of peripheral naive T cells, but may have some critical roles in special situations such as with aged mice.

## AA transporters and activation of T cells

Upon sensing a specific antigen by TCR on quiescent T cells, T cells are activated. Activated T cells proliferate rapidly and exert effector functions, such as cytokine production, which are largely dependent on the cellular metabolism to synthesize lipids, nucleic acids and proteins. Thus, once activated, T cells trigger a considerable metabolic switch, resulting in an increase in activities of the glycolytic pathway, pentose phosphate pathway and glutaminolysis, while decreasing FAO (Figure 1).^3, 4, 42^ Because of the increase in metabolic requirement, activated T cells need to consume large amounts of intracellular materials such as AAs and glucose; thus; they boost the rate of uptake of those nutrients by increasing the expression of transporters for glucose and AAs.

l-Leucine transportation by LAT1 increases during T-cell activation by PMA and ionomycin, compared with quiescent T cells.^43^ Also, the abundance of CD98 and LAT1 heterocomplex and the expression of LAT1 and CD98 mRNAs increases in activated T cells, compared with quiescent T cells.^43^ Although naive human primary T cells express an almost undetectable amount of LAT1 protein, activation of human primary T cells by anti-CD3 and anti-CD28 Abs markedly induces LAT1 abundance associated with activator protein-1 (AP-1) and nuclear factor-*κ*B (NF-*κ*B) signaling, which suggests that LAT1 is required for the full activation of T cells.^20^ JPH203 (a LAT1-specific inhibitor) or LAT1-specific siRNA treatment inhibits the uptake of l-leucine by human primary T cells, as well as attenuating the immunological functions of those T cells, such as the production of interferon-*γ* (IFN-*γ*), IL-4 and IL-17.^20^ Although naive CD8^+^ T cells do not effectively take up phenylalanine, activation of CD8^+^ T cells via TCR triggering with a cognate peptide significantly increases the transport of phenylalanine into those cells.^11^ Activated CD8^+^ T cells from mice immunized with *Listeria* also show enhanced phenylalanine uptake compared to naive T cells.^11^ Interestingly, activation CD8^+^ T cells increases the expression of *Slc3a2* and *Slc7a5*, and the abundance of LAT1 and CD98 proteins through calcineurin-regulated signaling pathways.^11^ *Slc7a5*-null CD4^+^ T cells do not respond to antigen receptor ligation, and *Slc7a5*-null CD8^+^ T cells also have a severe defect in their ability to respond to cognate antigen.^11^ *Slc7a5*-null OT-I CD8^+^ T cells do not undergo a proliferative expansion after antigen stimulation *in vivo*, indicating that Slc7a5 is essential for the CD8 T-cell-mediated immune responses.^11^ Mechanically, *Slc7a5*-null T cells are unable to activate mTORC1 signaling or express c-Myc protein; thus, these T cells have reduced glycolysis caused by a decrease in the expression of glucose transporter-1 (Glut1), glucose uptake and lactate output and decreased glutaminolysis caused by lowering glutamine and arginine uptake.^11^

Activated T cells have higher rates of uptake of glutamine (5–10-fold) compared with unstimulated T cells, and a deficiency in glutamine impairs the late events in T-cell activation, such as proliferation and cytokine secretion, although glutamine depletion has no effect on the initiation of the activation of T cells and their expression of T-cell surface markers CD69, CD25 and CD98.^44^ Activation of T cells through CD3 and CD28 induces the expression of the major glutamine transporters (SNAT-1 and SNAT-2), and relocation of those transporters from the cytoplasm to the cell surface.^44^ Activation of naive T cells with anti-CD3 plus anti-CD28 promotes a rapid increase in the uptake of glutamine, and prolonged T-cell activation further enhances the glutamine uptake, which is largely dependent on ASCT2 because anti-CD3- and anti-CD28-stimulated glutamine uptake is completely blocked in ASCT2-deficient T cells.^10^ Mechanically, proximal signals downstream of TCR and CD28 (CBM complex, composing of CARMA1, BCL10 and MALT1) mediate glutamine uptake induced by TCR and CD28 because a genetic deficiency in either CARMA1, BCL10 or MALT1 severely attenuates the anti-CD3 plus anti-CD28-induced glutamine uptake in T cells.^10^ The loss of CARMA1 markedly inhibits TCR- and CD28-stimulated ASCT2 mRNA expression, but has little effect on the expression of other AA transporters, including SNAT-1, SNAT-2, LAT1 and CD98.^10^ Also, CARMA1 physically interacts with ASCT2, which is required for ASCT2 to aggregate and colocalize rapidly with the TCR complex in response to TCR and CD28 stimulation.^10^ However, *Slc1a5*^+/+^ and *Slc1a5*^–/–^ T cells from young mice (6–7 weeks) display a similar ability to proliferate and produce IL-2 after stimulation by anti-CD3 and anti-CD28.^10^ Indeed, a deficiency in ASCT2 does not appreciably affect the TCR- and CD28-mediated activation of transcription factors, including NF-*κ*B, AP-1 and nuclear factor of activated T cells, and the TCR- and CD28-stimulated phosphorylation of signaling factors, including mitogen-activated protein kinases, ERK, JNK and p38, IκB kinase (IKK) and the IKK target I*κ*Ba.^10^ However, ASCT2 is necessary for TCR- and CD28-mediated activation of mTORC1 due to its effect on glutamine uptake.^10^

GAT-1 mRNA is detected in 50% of resting lymphocytes, whereas GAT-2 mRNA is not detected in those cells, but all activated lymphocytes express at least one of the two transporters.^17^ Further study has demonstrated that protein kinase C (PKC) has an important role in regulating GAT-1 expression by antigen-activated CD4^+^ T cells.^9^ Interestingly, although T cells from *Slc6a1*^−/−^ and WT mice have similar levels of [^3^H]thymidine incorporation after Con A stimulation, *Slc6a1*^−/−^ CD4^+^ T cells have more robust incorporation of [^3^H]thymidine after stimulation with anti-CD3 and anti-CD28 compared with WT CD4^+^ T cells.^9^ CD4^+^ T cells from *Slc6a1*^−/−^ mice also have higher IL-2 secretion and CD69 expression after stimulation with anti-CD3 and anti-CD28 compared with those cells from WT mice.^9^ GAT-1 negatively regulates CD4^+^ T-cell cycle entry from G_1_ to S phase by inhibiting the expression of G_1_–S phase proteins, such as cyclin A and CDK2, and by promoting the expression of p21^cip^ (an CDK inhibitor).^9^ GAT-1 deficiency interferes with apoptosis by enhancing the expression of antiapoptotic Bcl-2 family proteins.^9^ Mechanically, GAT-1 deficiency enhances the activity of PKC*θ* by regulating the translocation and phosphorylation of PKC*θ*, leading to phosphorylation of JNK and activation of the NF-*κ*B pathway to promote cell survival and cell division.^9^

Collectively, LAT1 and ASCT2 are positively related to the activation of T cells, whereas GATs are negatively associated with T-cell activation. These results indicate that AA transporters have critical roles in the activation of T cells.

## AA transporters and T-cell differentiation

**Figure 4:**
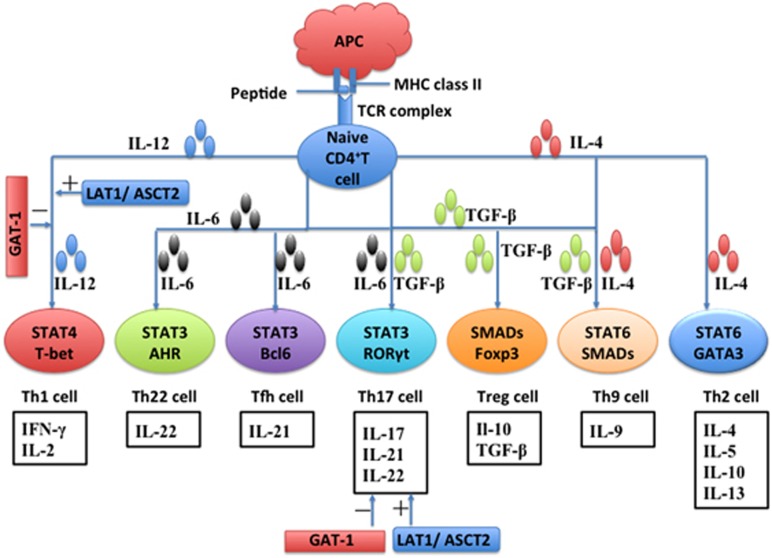
Influence of the cytokine environment on the differentiation of T-helper (Th) cells. Once activated by stimulation from T-cell receptor (TCR) complex signaling, the cytokine environment dictates Th cell differentiation. The prototypical cytokines and their corresponding signaling pathways that regulate the fate of each Th cell are depicted. There are also additional cytokine and signaling pathways that can influence Th cells. In mice, the development of Tfh cells depends on IL-6 and IL-21, whereas IL-12 and TGF-*β* signaling have critical roles in the development of Tfh cells in humans. LAT1 and ASCT2 are positively (+) associated with the differentiation of Th1 and Th17 cells, whereas GAT-1 is negatively (−) associated with the differentiation of Th1 and Th17 cells

The activated CD4^+^ T cells can differentiate into at least seven distinct states under a specialized cytokine environment, including Th1, Th2, Th9, Th17, Th22, Treg and T follicular helper (Tfh) cells, each with a specific phenotypic and unique functional characteristic (Figure 4). Th1 cells produce IL-2 and IFN-*γ*,^45^ and requires the cytokine IL-12, the master transcription factor TBX21 (T-box transcription factor) and the signaling transducer and activator of transcription-4 (STAT4) for its differentiation.^46^ Th2 cells produce IL-4, IL-5 and IL-13, as well as IL-10.^45, 47^ Available evidence shows that Th2 cell differentiation depends on IL-4 and is controlled by GATA3 (*trans*-acting T-cell-specific transcription factor) and STAT6.^48^ Th9 cells preferentially produce IL-9.^49, 50, 51^ Differentiation of Th9 cells and their release of IL-9 depend on IL-2, IL-4 and the transforming growth factor (TGF-*β*),^52^ and can be enhanced by IL-1.^49, 50^ IL-4 induced activation of STAT6 and TGF-*β*-mediated activation of SMADs, such as SMAD2, SMAD3 and SMAD4, are required for optimal differentiation of Th9 cells.^52^ Th17 cells secrete IL-17A, IL-17F, IL-21 and IL-22. TGF-*β*, IL-6, IL-1*β* and IL-23 promote Th17 cell differentiation through retinoic acid receptor-related orphan receptor-*γ*t, IFN-regulatory factor-4, aryl-hydrocarbon receptor (AHR) and STAT3.^53, 54, 55, 56^ Intriguingly, mTOR signaling also regulates Th17 cell differentiation and IL-17 gene expression.^57, 58, 59^ Th22 cells produce IL-22, but not IL-17 or IFN-*γ*.^60^ The development of Th22 cells from naive T cells requires stimulation of IL-6 and tumor necrosis factor-*α* (TNF-*α*) or antigens in the context of plasmacytoid dendritic cells, and depends on the AHR.^45^ Treg cells produce IL-10 and TGF-*β*.^45^ TGF-*β* is required for the generation of Tregs because TGF-*β* induces the expression of Foxp3 and SMADs signaling.^61, 62^ Tfh cells express CXCR5, PD-1, ICOS, CD40L, Bcl-6 and IL-21.^63^ In mice, IL-6 and IL-21 have critical roles in the development of Tfh cells, whereas IL-12 participates in the early phase of development of Tfh cells.^63, 64^ However, in humans, IL-12, IL-23 and TGF-*β* promote Tfh development by increasing the expression of multiple transcription factors in human naive CD4^+^ T cells, such as c-Maf and Batf, which are essential for Tfh development.^65, 66, 67^

*Slc7a5*-null CD4^+^ T cells cannot normally differentiate into Th1 or Th17 cells under the appropriate polarizing cytokines, but they respond normally to TGF-*β* and IL-2 to differentiate into Foxp3^+^ iTregs.^11^ *Slc7a5*^fl/fl^CD4-Cre mice have a defect in production of high-affinity IgG1 and affinity maturation of antibody specific for T-cell-dependent antigen nitrophenyl-OVA,^11^ indicating that Slc7a5 is also essential for the differentiation of Tfh cells. *Slc1a5*^–/–^ T cells have defects in Th1 and Th17 cell differentiation, but not in differentiation of Th2 cells or Foxp3^+^ Treg cells.^10^ Transfer of *Slc1a5*^–/–^ T cells to *Rag1*^–/–^ mice decreases IFN-*γ*^+^ Th1 cells, IL-17^+^ Th17 cells and IFN-*γ*^+^ IL-17^+^ DP T cells, when compared with those transferred with WT CD4^+^-naive T cells.^10^ Infection of the WT mice with *Listeria monocytogenes* induces a population of antigen-specific IFN-*γ*^+^ Th1 cells, whereas *Slc1a5*^–/–^ mice have profoundly reduced IFN-*γ*^+^ Th1 cells.^10^ Also, immunization of *Slc1a5*^+/+^ mice with a myelin oligodendrocyte glycoprotein peptide, along with injection with a pertussis toxin, leads to severe experimental allergic encephalomyelitis (EAE) clinical scores; however, the *Slc1a5*^–/–^ mice have much milder clinical EAE scores, and fewer IL-17-producing Th17 cells and IFN-*γ*-producing Th1 cells in the central nervous system under the same conditions.^10^ Mechanistically, the *Slc1a5*^–/–^ T cell failure to take up l-leucine leads to defects in mTORC1 signaling, c-Myc expression, Glut1 expression, glucose uptake, lactate secretion and glycolysis.^10^ *Slc6a1*^−/−^ mice have a higher expression of IFN-*γ*, TNF-*α*, IL-6, IL-23 and IL-17 mRNAs, compared to the WT mice.^16^ Mechanistically, *Slc6a1*^−/−^ mice have a greater expression of T-bet, STAT1 and pSTAT1, indicating that GAT-1 deficiency promotes the differentiation of IFN-*γ*-producing Th1 cells.^16^

In summary, LAT1 and ASCT2 positively regulate the differentiation of Th1 and Th17 cells, whereas GAT-1 negatively affects the differentiation of Th1 and Th17 cells. These findings strongly indicate the significant roles of AA transporters are in T-cell differentiation, especially for Th1 and Th17 cells. These findings also suggest that modulation of AA transporters could influence some T-cell-based immune diseases, such as EAE, IBD and asthma.

## AA transporters and T memory cells

Unlike activated or differentiated T cells, T memory (Tm) cells survive longer and undergo intermittent cell division. Based on homing and selectin molecule expression, effector cytokine production and location, Tm cells are subdivided into: T central memory cells, T effector memory cells, memory stem T cells, T-resident memory cells and follicular helper memory T cells.^68^ However, the homeostatic proliferation and survival of all subsets are dependent on stimulation from IL-15 and IL-7,^69, 70^ and all subsets metabolize glucose, fatty acids and AAs to generate ATP (Figure 1).^4, 71^ There is increasing evidence that the modulation of glucose metabolism or fatty acid metabolism affects the fate of Tm cells.^71, 72, 73^ The transportation of AAs by AA transporters or metabolism of AAs may also be critical in fate decisions of Tm cells. Interestingly, inhibition of mTOR, raptor or FK506-binding protein 12 by rapamycin treatment or RNA interference in antigen-specific CD8^+^ T cells has immunostimulatory effects on the generation of memory CD8^+^ T cells,^74^ which indicates that mTORC1 negatively the regulates CD8^+^ Tm cell fate decision, and that AAs may also negatively regulate CD8^+^ Tm cell functions. The percentage and number of memory CD4^+^ T cells are similar between young (6–7 weeks) *Slc1a5*^+/+^ and *Slc1a5*^–/–^ mice.^10^ However, *Slc1a5*^–/–^ with age of 5–6 months have a decreased percentage and number of memory CD4^+^ T cells, with a significant decrease in the population of CD44^hi^CD62L^lo^ memory T cells.^10^ Thus, AAs may influence Tm cell development and longevity in some special physiological situations. It is unknown whether AA metabolism has similar influences on memory CD4^+^ T cells and memory CD8^+^ T cells. The influence of AA metabolism or AA transportation on the fate decision of Tm cells requires further investigation in pathogen-infected or vaccine-immunized models.

## Mechanism for AA transporters in shaping T-cell biology

As discussed above, current evidence strongly highlights the importance of AA transporters in the activation of T cells, and differentiation of Th1 and Th17 cells. However, the molecular mechanism by which AA transporters regulate activation and differentiation of T cells is unknown. One of the main candidates is mTORC1 signaling, although other molecular signaling pathways remain to be discovered. Through the modulation of the cellular contents of AAs, AA transporters regulate the activation of mTORC1 signaling, which has a significant role in the activation of T cells, and differentiation of Th1 and Th17 cells.^75, 76^ For example, inhibition of mTORC1 signaling induces T-cell anergy even in the presence of signal 1 (TCR engagement) and 2 (costimulation).^77^ The inhibition of mTORC1 by rapamycin in CD4 T cells decreases the differentiation of Th1 and Th17 cells.^37^ The mTOR deficiency in T cells inhibits the differentiation of Th1, Th2 or Th17 cells from activated T cells.^37^ Deletion of RHEB in T cells inhibits the activation of mTORC1 signaling and differentiation of Th1 and Th17 cells.^78^ The mTORC1 signaling regulates Th17 differentiation through several pathways, including STAT3, hypoxia-inducible factor-1*α*, ribosomal S6 kinase-1 (S6K-1) and S6K2.^79^ Indeed, *Slc7a5*^–/–^ T cells and *Slc1a5*^–/–^ mice are defective regarding the activation of mTORC1.^10, 11^ Also, AA transporters have been associated with autophagy,^80, 81^ which has remarkable effects on the development, maturation, activation, differentiation and even memory of T cells;^82, 83^ thus, the influence of AA transporters on the activation and differentiation of T cells may be mediated via autophagy.

## Conclusions

**Figure 5:**
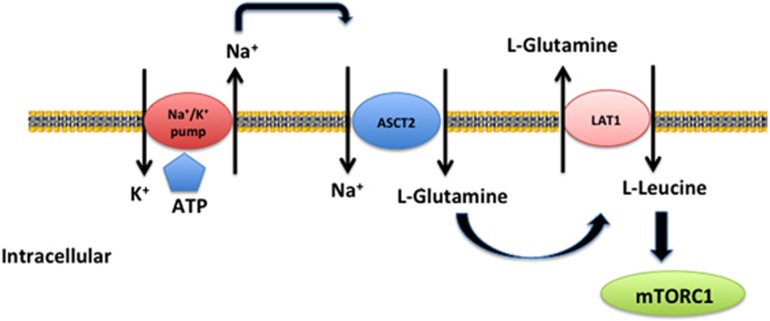
Mechanism of L-leucine uptake by ASCT2 and LAT1 transporters. Polarized Na^+^ transported by a Na^+^/K^+^ ATPase pump provides power to ASCT2, a Na^+^-dependent glutamine transporter, for cotransport of Na^+^ and glutamine. Concentrated glutamine is exchanged for L-leucine by an L-type AA transporter-1 (LAT1). L-leucine is an important activator of the mechanic target of rapamycin complex 1 (mTORC1) signaling

The cellular metabolism pathways highly shape the T-cell fate decision, including T-cell development, homeostasis, activation, differentiation and memory. The regulation of glucose metabolism and fatty acid oxidation in the fate decision of T cells is widely highlighted.^1, 2, 8^ AA transporters are also critical for the T-cell fate decision. This review has highlighted compelling evidence that AA transporters affect peripheral naive T-cell homeostasis, T-cell activation and differentiation, especially for Th1 and Th17 cells, and T cell memory (Figure 3). However, most investigations focus on LAT1 and ASCT2, and GAT-1 perhaps because LAT1 and ASCT2 are coupled with the transport of l-leucine (Figure 5),^29^ which is an important activator of mTOR signaling,^84^ and GAT-1 transports the GABA, a neurotransmitter. LAT1 and ASCT2 are positively related to T-cell activation, as well as Th1 and Th17 cell differentiation, whereas GAT-1 is negatively involved in the activation and differentiation of T cells (Table 1). However, the cellular and molecular mechanisms may be similar because l-leucine activates mTORC1 signaling in the cytoplasm,^84^ whereas GABA activates mTORC1 signaling through GABA receptors on the cell membrane (unpublished observation). GAT-1 terminates GABA signaling by mediating the translocation of GABA from the extracellular space into the intracellular space of cells. The mTORC1 signaling is of critical importance to the activation and differentiation of T cells;^59, 85, 86^ thus, the influence of AA transporters on the T-cell fate decision may largely depend on mTORC1 signaling, although other possible mechanisms remain to be known. It is also interesting to explore the expression of other AA transporters in T cells and regulation of the expressed AA transporters in the T-cell fate decision. Furthermore, as most research is conducted using conventional T cells, it will be of interest to uncover the importance of AA transporters on unconventional T cells, including CD1-restricted T cells, MR1-restricted mucosal- associated invariant T cells, MHC class Ib-reactive T cells and *γδ* T cells.^87^ We believe that understanding the influence of AA transporters in T-cell fate determination offers significant insights into T-cell-based immune diseases and opens up novel potential treatments to prevent and cure T-cell-based immune pathologies through modulation of the expression of AA transporters and the metabolism of AA in T cells.

## Figures and Tables

**Table 1:** Effect of mouse-related amino-acid transporter deficiency in T-cell fate decision

	*T-cell development*	*Naive T-cell homeostasis*	*T-cell activation*	*T-cell differentiation*	*T-cell memory*	*References*
*Slc7a5*^–/–^ mice	No	No	T-cell activation↓	Th1 and Th17 cells↓	Not available	^11^
*Slc1a5*^–/–^ mice	No	(1) For young mice^a^: No (2) For older mice^b^: CD4^+^ T cells↓ CD44^lo^CD62L^hi^ CD4^+^ T cells↑	Activation of mTORC1 in T cells^c^↓	Th1 and Th17 cells↓	(1) For young mice^a^: No (2) For older mice^b^: Memory CD4^+^ T cells↓	^10^
*Slc6a1*^–/–^ mice	No	No	IL-2 secretion and CD69 expression in T cells^c^ ↑	Th1 cells↑	Not available	^9, 71^

Abbreviations: IL, interleukin; mTORC1, mechanistic target of rapamycin complex 1; no, no effect; not available, the data are missing; Th, T-helper; ↑, increase; ↓, decrease.

a 6–7 weeks.

b 5–6 months.

c After anti-CD3 and anti-CD28 stimulation.

## Abbreviations

AA: amino acid
AHR: aryl-hydrocarbon receptor
AP-1: activator protein-1
ATP: adenosine triphosphate
DP: double-positive
DN: double-negative
EAE: experimental autoimmune encephalomyelitis
FAO: fatty acid oxidation
GABA: *γ*-aminobutyric acid
GAT: GABA transporter
Glut: glucose transporter
IFN-*γ*: interferon-*γ*
IKK: I*κ*B kinase
IL: interleukin
mTORC1: mechanistic target of rapamycin complex 1
NF-*κ*B: nuclear factor-*κ*B
OXPHOS: oxidative phosphorylation
PKC: protein kinase C
S6K: ribosomal S6 kinase
SNAT: sodium-dependent neutral amino-acid transporter
SP: single-positive
STAT: signaling transducer and activator of transcription
TCA: tricarboxylic acid
TCR: T-cell receptor
TGF: transforming growth factor
TNF: tumor necrosis factor
WT: wild type
