# Amino-acid transporters in T-cell activation and differentiation

**DOI:** 10.1038/cddis.2017.207

**Published:** 2017-05-04

**Authors:** Wenkai Ren, Gang Liu, Jie Yin, Bie Tan, Guoyao Wu, Fuller W Bazer, Yuanyi Peng, Yulong Yin

**Correction to:** *Cell Death and Disease* (2017) **8**, e2655; doi:10.1038/cddis.2016.222; published online 2 March 2017.

It has been noticed since publication of this article that the full names of the authors were omitted. The correct author list is as follows:

Wenkai Ren, Gang Liu, Jie Yin, Bie Tan, Guoyao Wu, Fuller W Bazer, Yuanyi Peng, Yulong Yin

